# Supplementary material for: Multi-omics analyses reveal that the gut microbiome and its metabolites promote milk fat synthesis in Zhongdian yak cows
Source: PeerJ. 2022 Dec 2;10:e14444. doi: 10.7717/peerj.14444 (PMC9744170; doi:10.7717/peerj.14444)
Supplement: Supplemental Information 13 [file peerj-10-14444-s013.zip › Web_Report/HTML/H_vs_L.KEGG_diff_anno.xls.html]

H\_vs\_L.KEGG\_diff\_anno.xls


## H\_vs\_L.KEGG\_diff\_anno.xls

| #ID | name | H5 | H4 | H2 | H6 | H1 | H3 | L1 | L5 | L4 | L2 | L6 | L3 | H\_Mean | L\_Mean | Fold\_change | log2FC | Pvalue | VIP | regulated | KEGG\_pathway\_annotation |
| --- | --- | --- | --- | --- | --- | --- | --- | --- | --- | --- | --- | --- | --- | --- | --- | --- | --- | --- | --- | --- | --- |
| pos\_1 | D-erythro-Dihydrosphingosine | 0.00016750424081482 | 0.000254417617725337 | 0.000280516099889959 | 0.000200086248543327 | 0.000143373613404701 | 0.000224718909490409 | 0.000130812981686961 | 0.000157863228276111 | 0.000127429588182838 | 0.000139869306485217 | 0.000141074891906182 | 0.000124289239886146 | 0.000211769454978092 | 0.000136889872737243 | 0.646409902463998 | -0.596313270704428 | 0.0156423085365592 | 2.28980826228015 | down | Sphingolipid signaling pathway(ko04071);;Sphingolipid metabolism(ko00600);;Metabolic pathways(ko01100) |
| pos\_1073 | Neolinustatin | 8.65184137415296e-06 | 7.88622271234797e-06 | 9.85186204859414e-06 | 8.04562355504655e-06 | 5.96503890331222e-06 | 6.88259674573533e-06 | 1.27405048068724e-05 | 9.8469071217803e-06 | 8.4786020981128e-06 | 1.11270187582944e-05 | 9.08477593083803e-06 | 1.46742998177055e-05 | 7.88053088986486e-06 | 1.09920180889339e-05 | 1.39483218104896 | 0.471514439782077 | 0.0233300137844173 | 2.28950696821559 | up | -- |
| pos\_108 | RHOIFOLIN | 9.23534477713109e-06 | 2.0370241796344e-06 | 1.82688113090293e-05 | 1.09351704987591e-05 | 3.78775372116092e-06 | 3.57014084103671e-05 | 1.71236608976507e-05 | 4.17430306845687e-05 | 3.84908284112322e-05 | 8.49582130333884e-05 | 6.2976908725647e-05 | 7.69590820373692e-05 | 1.33275854826803e-05 | 5.37086206316427e-05 | 4.02988378513415 | 2.40736375921449 | 0.0100836609418814 | 2.51792413422118 | up | -- |
| pos\_1141 | Isovalerylglutamic acid | 2.03288102216096e-05 | 1.77626953626292e-05 | 2.5607171467383e-05 | 1.50831721446601e-05 | 1.72535810088484e-05 | 8.31919499750707e-06 | 3.6666681758369e-05 | 3.35311177847392e-05 | 2.53988822599635e-05 | 4.73858259215339e-05 | 3.1145888413144e-05 | 1.32588457165065e-05 | 1.73924375337729e-05 | 3.12312069757094e-05 | 1.79567739801072 | 0.821985341430841 | 0.0313728760477961 | 2.17392310639798 | up | -- |
| pos\_1143 | PE(15:0/20:2(11Z,14Z)) | 6.36832831399463e-06 | 1.71658318304358e-06 | 6.70664798489389e-06 | 5.25051210775874e-06 | 7.45195400458137e-06 | 1.85788846995226e-06 | 9.18290147686821e-07 | 5.13156736766791e-06 | 1.30459372443255e-06 | 2.1367799335147e-06 | 1.36535314825349e-06 | 1.34561322289143e-06 | 4.89198567737074e-06 | 2.03369959074115e-06 | 0.415720675583455 | -1.29779813212204 | 0.0440446544853637 | 1.91196599865638 | down | -- |
| pos\_1150 | 1-Acetoxy-2-hydroxy-16-heptadecen-4-one | 0.000151575413079437 | 0.000255722475878777 | 0.000185180540263676 | 0.000214015643375558 | 0.000207613115783886 | 0.000240533712572556 | 0.000268864722093845 | 0.000267203344635357 | 0.000184649679281645 | 0.000280175496118886 | 0.000278484596259226 | 0.000313248453386198 | 0.000209106816825648 | 0.00026543771529586 | 1.26938814968036 | 0.346574136619578 | 0.0365923216846916 | 2.00982245514272 | up | -- |
| pos\_1180 | Ustiloxin B | 0.000376152762860401 | 0.000112274177783131 | 0.000320636273840527 | 0.000211077003869773 | 0.000326569621825361 | 3.22435747774862e-07 | 1.41789851109209e-06 | 1.23058559592231e-05 | 0.000134511448926682 | 8.73226451032093e-07 | 2.672021042301e-07 | 8.35321758639335e-07 | 0.000224505379321161 | 2.50351589518165e-05 | 0.111512512651213 | -4.88222659615547 | 0.0183477653817877 | 2.29697191702385 | down | -- |
| pos\_1248 | Cerebroside B | 2.09961928551289e-06 | 1.07254822091939e-06 | 1.91119367320607e-06 | 5.61763743287234e-06 | 7.27937688743303e-06 | 2.80738716624481e-06 | 1.09353927396022e-06 | 9.35717321816451e-07 | 0 | 0 | 8.4742868203384e-07 | 2.08240165138295e-06 | 3.46462711103142e-06 | 8.2651448819891e-07 | 0.238557992450984 | -8.77230315488751 | 0.0446680502600719 | 2.03886743215265 | down | -- |
| pos\_1249 | Quinquenoside III | 4.97797653238683e-06 | 3.6449387498287e-06 | 4.41150192560898e-06 | 5.72121314580859e-06 | 2.62968983836385e-06 | 5.63090614321353e-06 | 5.45775415226494e-06 | 8.55070703871328e-06 | 7.24801579382881e-06 | 6.67223761888877e-06 | 5.65853649207721e-06 | 5.15841637454096e-06 | 4.50270438920175e-06 | 6.45761124505233e-06 | 1.43416282457689 | 0.546693388008443 | 0.0220401459770514 | 2.17887961209206 | up | -- |
| pos\_1270 | PC(14:1(9Z)/22:5(4Z,7Z,10Z,13Z,16Z)) | 6.1357186610487e-06 | 2.08103996091174e-06 | 6.88064879033784e-06 | 1.77380669725318e-06 | 6.87083562982464e-06 | 1.18578102874268e-05 | 2.27158870131552e-06 | 7.85798908996056e-07 | 1.58358658921546e-06 | 2.39117913289514e-06 | 0 | 4.35514326331569e-06 | 5.93331000446715e-06 | 1.89788276595631e-06 | 0.319869139574269 | -5.1702458135805 | 0.0451902691522032 | 2.02303422188602 | down | -- |
| pos\_1347 | 1(10),11-Eremophiladiene-2,9-dione | 5.46529717413635e-06 | 5.80958631703303e-06 | 5.38725000230506e-06 | 5.36196926011316e-06 | 4.24099807436897e-06 | 5.88934074340801e-06 | 5.73398984873798e-06 | 6.20198315186751e-06 | 5.95688276336644e-06 | 5.39266050009931e-06 | 6.90302407684911e-06 | 6.8049647913759e-06 | 5.35907359522743e-06 | 6.16558418871604e-06 | 1.15049440526565 | 0.204680437587385 | 0.0405049492731453 | 1.92112388079339 | up | -- |
| pos\_137 | 2,6,6-Trimethyl-2-cyclohexene-1,4-dione | 0.000179773753278698 | 0.000109162126041124 | 0.000177294662685662 | 0.000137010659799997 | 0.000173893928184645 | 2.24096864328154e-05 | 1.97511558758124e-05 | 5.0594031133571e-05 | 0.000131901885625438 | 2.41227576760009e-05 | 2.43181109419071e-05 | 2.83048422175909e-05 | 0.000133257469403824 | 4.64987972450534e-05 | 0.348939518760809 | -1.62154834975248 | 0.019368451282156 | 2.18180020658806 | down | -- |
| pos\_1370 | FMNH2 | 1.3822956366106e-05 | 2.74876998979499e-06 | 1.41158741298001e-05 | 6.66522024259159e-06 | 1.85471589702265e-05 | 0 | 1.8953720964621e-07 | 5.08894023222634e-07 | 4.52544860509124e-06 | 3.46808654564704e-07 | 2.7550334907704e-07 | 9.17553807195752e-08 | 9.31666328308653e-06 | 9.896578703869e-07 | 0.106224496938032 | -0.296266625005515 | 0.0372508075170043 | 2.14621619463489 | down | Metabolic pathways(ko01100);;Riboflavin metabolism(ko00740) |
| pos\_1376 | Brassicoside | 9.71768486525661e-06 | 7.98012419853088e-06 | 1.16962725187996e-05 | 7.19593711026315e-06 | 1.0054481960694e-05 | 0 | 0 | 7.16181342852175e-07 | 6.89429874645566e-06 | 0 | 0 | 0 | 7.77408344225737e-06 | 1.26841334821797e-06 | 0.163159214541394 | -13.417457446312 | 0.0111360900545616 | 2.31779383227658 | down | -- |
| pos\_1381 | Araliasaponin III | 3.15883599279883e-05 | 4.04423943027185e-05 | 3.63332145908761e-05 | 4.86669122471633e-05 | 2.49349552505218e-05 | 4.73268578129351e-05 | 4.95540982352512e-05 | 4.55691760025956e-05 | 4.9075064340246e-05 | 4.15491509500115e-05 | 5.44700190339456e-05 | 4.9763153662264e-05 | 3.82154490220338e-05 | 4.8330110370719e-05 | 1.26467466973509 | 0.371181941092248 | 0.0441686747092118 | 2.04748890170849 | up | -- |
| pos\_1432 | Goshonoside F3 | 7.42032617588857e-05 | 7.0393814916351e-05 | 7.65688896979071e-05 | 7.12186968812788e-05 | 5.59293455279314e-05 | 8.25044088866702e-05 | 6.17093190887472e-05 | 7.04236711333495e-05 | 5.44749631189395e-05 | 6.05704270500826e-05 | 5.95658368497939e-05 | 6.50439455868172e-05 | 7.1803069611504e-05 | 6.19646938046216e-05 | 0.862981125178719 | -0.207061117475236 | 0.0485318624253607 | 1.8332207990078 | down | -- |
| pos\_1433 | 3-Hydroxy-1-phenyl-1-hexadecanone | 0.000351826523135804 | 0.000254614809597739 | 0.000235444025996873 | 0.000367281203785349 | 0.000518425994795781 | 0.000301407774482569 | 0.000233289164279591 | 0.000309627291370091 | 0.000142679032145728 | 0.000159529846376317 | 0.000248133412945093 | 0.000241269437658232 | 0.000338166721965686 | 0.000222421364129175 | 0.657726942604792 | -0.603381937484322 | 0.0443674428604515 | 2.06897841574699 | down | -- |
| pos\_1434 | Salviaflaside | 0.00105425588001819 | 0.000593033460751 | 0.000973770939158533 | 0.000695105137800081 | 0.000992764592237792 | 2.34873176818525e-05 | 1.74591784618121e-05 | 0.000142304531724802 | 0.000683988168007077 | 1.786934163129e-05 | 1.93499548715374e-05 | 4.16647037278124e-05 | 0.000722069554607908 | 0.000153772646404055 | 0.212960988900239 | -3.10343619686685 | 0.0161574333228049 | 2.24631804078918 | down | -- |
| pos\_1475 | 1-Hydroxy-1-phenyl-3-octadecanone | 0.000125492732283598 | 0.000166621446886194 | 9.80306290752893e-05 | 0.000155859884786584 | 0.000105164732454102 | 0.000121108135287998 | 0.000151906027425918 | 0.00015254499069377 | 0.000155001770446212 | 0.000183840474654909 | 0.000201857729324394 | 0.000147274719374718 | 0.000128712926795628 | 0.000165404285319987 | 1.28506350867632 | 0.378387143246274 | 0.0295583513896161 | 2.06517231457104 | up | -- |
| pos\_1502 | Ganglioside GA2 (d18:1/16:0) | 7.99604501454314e-05 | 9.96551733646186e-05 | 0.000155476740368967 | 8.16356293165536e-05 | 0.000117592607182848 | 0.000124430243566759 | 0.000199457001047959 | 0.000145031723297999 | 0.000139928231633722 | 0.00011260425675157 | 0.000141539632725705 | 0.000144527328613111 | 0.000109791807324196 | 0.000147181362345011 | 1.34054959046634 | 0.442577304442521 | 0.046942050794908 | 1.86003895674376 | up | -- |
| pos\_1509 | PE(22:5(4Z,7Z,10Z,13Z,16Z)/dm18:0) | 2.67611363850322e-06 | 1.24640828015713e-06 | 3.92383898305321e-06 | 3.55704313657996e-06 | 5.1285710602548e-06 | 0 | 9.50334006318637e-07 | 0 | 0 | 0 | 9.17865890860539e-07 | 7.97310652938733e-07 | 2.75532918309139e-06 | 4.44251758352985e-07 | 0.161233641729352 | -8.82684528745938 | 0.0281643594987611 | 2.16543271511476 | down | -- |
| pos\_1534 | 2-Methyl-3-(2-pentenyl)-2-cyclopenten-1-one | 3.45079780121485e-05 | 3.8198469205698e-05 | 3.77794381751597e-05 | 3.62583770274909e-05 | 3.63811072416151e-05 | 3.25636457174487e-05 | 3.851614028093e-05 | 3.85132611496119e-05 | 4.01323976587732e-05 | 3.83516385775183e-05 | 3.8820388195475e-05 | 3.64893783176681e-05 | 3.59481692299268e-05 | 3.84705340299961e-05 | 1.07016671096478 | 0.0993921784347087 | 0.0341616947442768 | 2.06258561770893 | up | -- |
| pos\_1549 | 4-Methyl-2-pentyl-1,3-dioxolane | 0.000492369989726271 | 0.000615208377537651 | 0.000367570032899753 | 0.000605351347258427 | 0.000367409891145014 | 0.000527167251586901 | 0.00063395215042419 | 0.000575665738729368 | 0.000634031674178458 | 0.0006787652795462 | 0.000754160362633074 | 0.000521891276911758 | 0.000495846148359003 | 0.000633077747070508 | 1.27676245780203 | 0.373905420841716 | 0.035087027432012 | 2.00590066389473 | up | -- |
| pos\_1572 | (R)-Laudanidine | 0.000286487559230178 | 0.000125715761802576 | 0.000224995017531134 | 0.000210856228464749 | 0.000420899549046064 | 2.11460843023292e-05 | 2.08085554706771e-05 | 4.83642476357147e-05 | 0.000119117176752181 | 2.27918021040984e-05 | 2.17310713916936e-05 | 2.56210229887475e-05 | 0.000215016700062838 | 4.30723127238521e-05 | 0.200320778391931 | -2.21454466379704 | 0.026058649507241 | 2.27801251368211 | down | -- |
| pos\_1633 | Tetracosahexaenoic acid | 0.000213731043092909 | 0.000165046142573777 | 0.000183401989900968 | 0.000168610103711334 | 0.000159379367504349 | 0.000154698471403379 | 0.000193238291807224 | 0.000193225132662388 | 0.000225713467567169 | 0.000318394322657193 | 0.000234845069770126 | 0.000232211492262035 | 0.000174144519697786 | 0.000232937962787689 | 1.33761293890807 | 0.407304927984015 | 0.0245999985554968 | 2.30449557623729 | up | -- |
| pos\_1653 | 3,8-Dihydroxy-6-methoxy-7(11)-eremophilen-12,8-olide | 8.88643537148372e-06 | 4.53849361562092e-06 | 8.57988856873519e-06 | 5.93706403160147e-06 | 6.06324947033425e-06 | 1.26511166039424e-06 | 2.15225514203341e-06 | 1.49648939819358e-06 | 5.84955415754121e-06 | 1.60317723886771e-06 | 1.19583604818397e-06 | 1.20357869491511e-06 | 5.87837378636163e-06 | 2.2501484466225e-06 | 0.382784172698077 | -1.41697349840234 | 0.0271142466505614 | 2.06994246045655 | down | -- |
| pos\_1662 | Linoleoyl ethanolamide | 1.01236618399633e-05 | 1.24195061970836e-05 | 1.1984695330253e-05 | 1.10536026872802e-05 | 1.04558333504073e-05 | 1.09375573644376e-05 | 7.87724213085164e-06 | 9.17628165015446e-06 | 1.02609217450509e-05 | 1.07733449820655e-05 | 8.79488281743722e-06 | 7.73941146716757e-06 | 1.11624761282375e-05 | 9.10368079878788e-06 | 0.815561054214349 | -0.301374140246885 | 0.00876152179642622 | 2.26848435833624 | down | -- |
| pos\_1676 | Ginsenoyne N | 0.003703951013433 | 0.0043546032465507 | 0.00423234147582069 | 0.00403273216633599 | 0.00455336917297641 | 0.00378564900452259 | 0.00540457427028605 | 0.00485232555865995 | 0.00484703067662243 | 0.00484202130750251 | 0.00439865903996059 | 0.00442753570163052 | 0.00411044101327323 | 0.00479535775911034 | 1.16662853052152 | 0.22280677910743 | 0.00690168617740647 | 2.35013744138777 | up | -- |
| pos\_169 | 1,5-Octadien-3-one | 0.000119241974338063 | 7.12925741751359e-05 | 0.000116451307149837 | 8.96296971051631e-05 | 0.000119906615485683 | 8.84149593907544e-06 | 5.50319443792603e-06 | 2.60203497421662e-05 | 8.62031203040669e-05 | 5.9508560456319e-06 | 8.1738278484605e-06 | 1.18676738753736e-05 | 8.75606106988262e-05 | 2.39531703756042e-05 | 0.273561024579803 | -2.28139897388003 | 0.016933031333629 | 2.22085377716555 | down | -- |
| pos\_171 | 6-Methyl-3,5-heptadien-2-one | 0.000154594245890527 | 7.36182320650965e-05 | 0.000152712851748616 | 0.000106074078167348 | 0.00015727986599281 | 9.02602190435668e-06 | 6.56725330852377e-06 | 2.62328037146796e-05 | 8.77134152610845e-05 | 1.20708137270274e-05 | 9.95783215804234e-06 | 1.09637230685815e-05 | 0.000108884215961459 | 2.55843068729899e-05 | 0.234968003829368 | -2.28032600798234 | 0.0170028561167941 | 2.2600951527229 | down | -- |
| pos\_1720 | N-arachidonoylglycinate | 4.84866143431312e-06 | 5.87724486977586e-06 | 5.27837810689005e-06 | 3.90289099711463e-06 | 2.01990518871537e-06 | 4.33462836762712e-06 | 7.90830401737774e-06 | 1.02711284390286e-05 | 9.85687455618273e-06 | 1.23017882522485e-05 | 8.21921502245616e-06 | 9.73017249343252e-06 | 4.37695149407269e-06 | 9.71458046345438e-06 | 2.21948552014112 | 1.20995387407135 | 0.000100579690172435 | 2.98025573847469 | up | -- |
| pos\_177 | Sorbaldehyde | 9.63911449398946e-05 | 5.58567045964917e-05 | 9.40567269680116e-05 | 7.27452671784096e-05 | 9.29527433516892e-05 | 7.84459323925632e-06 | 6.11422266290188e-06 | 2.28107930156945e-05 | 6.93662648711207e-05 | 5.84347274634726e-06 | 8.20323888911842e-06 | 1.10735785570469e-05 | 6.99745300456255e-05 | 2.05685951237049e-05 | 0.293944026637887 | -2.0588011337217 | 0.018469076305247 | 2.19487286937673 | down | -- |
| pos\_1781 | 1-Caffeoyl-5-feruloylquinic acid | 2.86815025615071e-05 | 2.15489884073793e-05 | 3.29876639221123e-05 | 2.10782468048016e-05 | 2.9021360726673e-05 | 7.5777366337567e-07 | 3.46742107997454e-06 | 8.17103702515063e-06 | 2.43405496352548e-05 | 5.82606866402045e-06 | 2.63788816008932e-06 | 2.67110300241194e-06 | 2.23459226809748e-05 | 7.85234459448361e-06 | 0.351399434545124 | -1.39716716562842 | 0.0340602332448421 | 2.01153763752939 | down | -- |
| pos\_1820 | Edulisin I | 0.000233921772395635 | 0.000304810735446982 | 0.000261108258228178 | 0.000241912534329992 | 0.000293776266539317 | 0.000102898963993551 | 8.86960650881635e-05 | 0.000131180981887268 | 0.000133518097459212 | 0.000208691657446914 | 0.000138510136732917 | 9.46055752621127e-05 | 0.000239738088488942 | 0.000132533752312765 | 0.552827267240339 | -0.833687047784428 | 0.0141360014653515 | 2.23549160664019 | down | -- |
| pos\_1834 | (Z,Z)-2,9,16-Heptadecatriene-4,6-diyn-8-ol | 1.01149832115804e-05 | 3.76702358034263e-06 | 6.25310223604237e-06 | 5.11569007523731e-06 | 2.64768517463062e-06 | 3.4404814179057e-07 | 3.30834818012272e-07 | 4.1644510230494e-07 | 6.07781045097487e-07 | 3.41886783988525e-07 | 7.31359577874189e-07 | 3.76551337480178e-07 | 4.70708873660398e-06 | 4.67476444126265e-07 | 0.0993132847679296 | -2.85059922792063 | 0.0266710528319278 | 2.23279958692099 | down | -- |
| pos\_1857 | DG(18:4(6Z,9Z,12Z,15Z)/22:2(13Z,16Z)/0:0) | 0.000286548118717452 | 1.06989384326274e-05 | 1.91728127179022e-05 | 0.000106611725781386 | 0.000207241032433704 | 1.60481104108214e-05 | 9.7349183604498e-05 | 0.000269232889687937 | 0.00028077190391138 | 0.000440589993149029 | 0.000337675903058598 | 0.000241362338933146 | 0.000107720123082316 | 0.000277830368724098 | 2.57918725651465 | 2.27169475512538 | 0.0279818605634281 | 2.04862668358881 | up | -- |
| pos\_1877 | Albigenic acid | 2.96926505206843e-05 | 3.75527428666188e-05 | 3.84090468112887e-05 | 3.22957082162594e-05 | 4.19735032777377e-05 | 2.19137536897679e-05 | 3.54110472094531e-05 | 4.73116385046316e-05 | 6.57971778770263e-05 | 6.11479702723905e-05 | 5.67899374111139e-05 | 6.00754296054173e-05 | 3.36395675637261e-05 | 5.44222001466721e-05 | 1.61780320283772 | 0.696001172561459 | 0.00445502646584551 | 2.53716829604791 | up | -- |
| pos\_1878 | (±)-Rollipyrrole | 6.40538748190744e-06 | 1.1630285569869e-06 | 2.64195719880272e-06 | 2.16521993632177e-06 | 2.79921133933176e-06 | 1.49592524057624e-06 | 1.20814007541974e-06 | 1.58349182974329e-05 | 1.09936800026234e-05 | 2.10830993248764e-05 | 1.06088306992744e-05 | 7.70444353794624e-06 | 2.7784549589878e-06 | 1.12388519895955e-05 | 4.0450006048289 | 1.83132929629747 | 0.0274018692974361 | 2.28202398133804 | up | -- |
| pos\_1908 | Calendasaponin B | 3.46795220020815e-05 | 3.20705248359784e-05 | 4.15257327742404e-05 | 2.65541554270807e-05 | 6.62961648231401e-05 | 4.12751908370587e-05 | 2.94412644439757e-05 | 1.88076684602546e-05 | 2.38587833094869e-05 | 2.31711022761307e-05 | 1.52433673859452e-05 | 1.81368316413224e-05 | 4.04002151165966e-05 | 2.14431695861859e-05 | 0.530768698243316 | -0.884893943186183 | 0.0188326992772981 | 2.38553878322572 | down | -- |
| pos\_1917 | 3'-Deaminofusarochromanone | 0.000129007367765583 | 0.000106436028235151 | 9.364225030884e-05 | 0.000119197334859798 | 0.000119273773592025 | 9.52876515081652e-05 | 9.38370821228119e-05 | 0.000154289162044842 | 0.000150717419275161 | 0.000199888085784674 | 0.000153731121093064 | 0.000145279763124455 | 0.000110474067711594 | 0.000149623772240835 | 1.35437913476171 | 0.413914481174565 | 0.035821514755463 | 2.14124248575738 | up | -- |
| pos\_1927 | Glutamyl-Lysine | 0.0110423999920611 | 0.00679009668278375 | 0.00645656766729617 | 0.00795290404372468 | 0.00824769522277654 | 0.0108089688380562 | 0.00528452113251467 | 0.00576137187116136 | 0.00728689010436488 | 0.00515252143144847 | 0.00599918616472132 | 0.00682219592238915 | 0.00854977207444974 | 0.00605111443776664 | 0.707751549991593 | -0.479173539441435 | 0.0249566232133843 | 2.29533463207272 | down | -- |
| pos\_1999 | N-Despropyl-rotigotine | 1.01179920859605e-06 | 1.07369833522837e-06 | 9.72647091982189e-07 | 1.19858386841974e-06 | 1.13302883446215e-06 | 1.52436126276636e-06 | 1.83344726643403e-05 | 1.13950472584905e-05 | 7.48235544717182e-06 | 1.16409051919916e-05 | 6.1038881716978e-06 | 1.48854435387125e-06 | 1.15235310024248e-06 | 9.40753551459388e-06 | 8.16376118796778 | 2.70097601435873 | 0.0170746890557082 | 2.40503041727147 | up | -- |
| pos\_2025 | Nb-p-Coumaroyltryptamine | 1.1034877348056e-05 | 8.78672976298007e-06 | 1.37123984691986e-05 | 1.01415950170239e-05 | 1.12427478181628e-05 | 2.50639890330059e-05 | 6.7879092587032e-06 | 2.23259017457921e-05 | 2.78406424707356e-05 | 2.72989025993846e-05 | 3.21154285119862e-05 | 2.73293814279885e-05 | 1.33303895747379e-05 | 2.3949694335765e-05 | 1.79662373717506 | 0.78894456406482 | 0.0397700142167762 | 1.98859106936626 | up | -- |
| pos\_2027 | (3b,6a,12b,17a,20S)-Dammar-24-ene-3,6,12,17,20-pentol 20-[glucosyl-(1->2)-[rhamnosyl-(1->6)]-glucoside] 6-xyloside | 9.57506131970197e-06 | 7.98635256699231e-06 | 6.17683724826478e-06 | 7.8984520057108e-06 | 7.35298566895993e-06 | 1.16113123400317e-05 | 7.86520135364678e-06 | 1.24238178014197e-05 | 1.50682523960723e-05 | 1.62495361449432e-05 | 1.47308210407475e-05 | 1.38339070780034e-05 | 8.43350019161025e-06 | 1.33619226358055e-05 | 1.58438635586895 | 0.656023976800697 | 0.0084011760943998 | 2.38739650148048 | up | -- |
| pos\_2057 | N5-Acetyl-N2-gamma-L-glutamyl-L-ornithine | 3.27381035799066e-05 | 2.45607539677717e-05 | 3.57418213349484e-05 | 3.92927062334747e-05 | 2.87823582460224e-05 | 3.27700821866356e-05 | 3.3445086941365e-05 | 3.85696711271216e-05 | 4.81189069811282e-05 | 4.82122720394676e-05 | 4.09024054913717e-05 | 4.33176542479318e-05 | 3.23143042581266e-05 | 4.2094332804731e-05 | 1.30265322961873 | 0.385940309759317 | 0.0112572841324195 | 2.45516430544514 | up | -- |
| pos\_2122 | 6,7-dihydro-12-epi-LTB4 | 5.18263345575681e-05 | 5.56622102735305e-05 | 6.61860494865881e-05 | 5.74295970930128e-05 | 5.23114122661673e-05 | 6.13334743385158e-05 | 4.62680251228542e-05 | 8.13568125371573e-05 | 9.04131185582185e-05 | 9.84123655554039e-05 | 8.71764070639232e-05 | 8.37076014029753e-05 | 5.74581796692304e-05 | 8.12223883734221e-05 | 1.41359139535911 | 0.466073537083714 | 0.0222809541153942 | 2.36740127019597 | up | -- |
| pos\_2142 | 3-carboxy-4-methyl-5-propyl-2-furanpropanoate | 0.000628584383162663 | 0.000304212906614103 | 0.000652552452454275 | 0.000411832961233399 | 0.000578744580310164 | 3.61347431796016e-05 | 3.94080373704698e-05 | 9.65743642621704e-05 | 0.000313834577719133 | 3.91212298098121e-05 | 4.0204140868438e-05 | 3.9624726298035e-05 | 0.000435343671159034 | 9.4794512721343e-05 | 0.217746389809613 | -2.3029225795434 | 0.0152085452637744 | 2.29988492959908 | down | -- |
| pos\_2158 | PE(22:6(4Z,7Z,10Z,13Z,16Z,19Z)/24:1(15Z)) | 3.58098143223364e-05 | 7.07315417162826e-05 | 4.87096694516003e-05 | 5.31704747701709e-05 | 4.40548327089505e-05 | 5.33129361740034e-05 | 6.61846885796234e-05 | 9.73023897994007e-05 | 6.21163889855879e-05 | 0.000129902559714265 | 0.000105969454534642 | 6.49979019139092e-05 | 5.09648781905574e-05 | 8.7745563921238e-05 | 1.72168691531368 | 0.756324359054664 | 0.021048534380251 | 2.31845515076388 | up | -- |
| pos\_2192 | PS(DiMe(11,5)/DiMe(13,5)) | 7.85400181601917e-05 | 5.32899373656628e-05 | 0.000110663171587337 | 7.82204022482555e-05 | 0.000135497673892434 | 0.00010003151668548 | 5.45468381921281e-05 | 4.64403051174801e-05 | 4.8052170767097e-05 | 6.44341042307777e-05 | 4.77398796837856e-05 | 7.07693234307901e-05 | 9.27071199898935e-05 | 5.53304369036764e-05 | 0.596830501365033 | -0.70198333470419 | 0.0232876641041064 | 2.2031385427726 | down | -- |
| pos\_2196 | Pectenotoxin 3 | 2.18155619504515e-05 | 1.25738260038678e-05 | 1.79916144750811e-05 | 1.50300876139465e-05 | 1.33596983046455e-05 | 1.45715430456606e-05 | 2.35501092400871e-05 | 2.69186108559527e-05 | 3.14564743524474e-05 | 1.9475174880179e-05 | 2.44957197869696e-05 | 3.5921053755078e-05 | 1.58903885656088e-05 | 2.69695238117856e-05 | 1.69722242476531 | 0.760781639284 | 0.00406357117033221 | 2.51836950118873 | up | -- |
| pos\_2217 | 7-Chloro-3,4',5,6,8-pentamethoxyflavone | 4.12308757833539e-05 | 1.74835049950224e-05 | 4.35368822175474e-05 | 2.40837821661211e-05 | 3.50411173562552e-05 | 3.25983293520926e-07 | 1.20898727267271e-06 | 5.93522047948137e-06 | 2.19966285201795e-05 | 6.98450245997044e-06 | 2.30131437801194e-06 | 3.63275017897762e-07 | 2.69503576353035e-05 | 6.46498802136895e-06 | 0.23988505491668 | -2.19891968641324 | 0.0278258267492436 | 2.10344586114265 | down | -- |
| pos\_2218 | Bilirubin diglucuronide | 1.52214112274094e-05 | 1.52361398621242e-06 | 9.24526602173704e-06 | 6.01225476192585e-06 | 1.2398832645844e-05 | 0 | 0 | 0 | 2.7848740219841e-06 | 0 | 0 | 0 | 7.40022977385479e-06 | 4.64145670330683e-07 | 0.0627204403801788 | -16.9270215224952 | 0.0362649406252354 | 2.17558314006078 | down | Porphyrin and chlorophyll metabolism(ko00860);;Metabolic pathways(ko01100) |
| pos\_2221 | Matsutakic acid A | 0.000201940087940583 | 0.000235405101099329 | 0.000233134208608594 | 0.000216823628662381 | 0.000242659901320921 | 0.000206720193916594 | 0.00027238371885424 | 0.000265439672753517 | 0.000272070812398608 | 0.000285105912362693 | 0.000245726830162415 | 0.000231674805601176 | 0.000222780520258067 | 0.000262066958688775 | 1.17634593179511 | 0.234215085953931 | 0.00409964376792742 | 2.49525409570738 | up | -- |
| pos\_2226 | 3-Oxooctadecanoic acid | 9.96527197762701e-05 | 7.68478885894957e-05 | 7.89418944507548e-05 | 6.5205099775442e-05 | 7.53995883283602e-05 | 7.30218372098261e-05 | 8.25813885034055e-05 | 9.88364589332852e-05 | 0.000115102788203717 | 0.000186815981350436 | 0.000132565861306094 | 0.000103503562631583 | 7.81781713550248e-05 | 0.00011990100682142 | 1.53368906874174 | 0.57865456647836 | 0.0381426492067514 | 2.17664709238761 | up | -- |
| pos\_2239 | CDP-DG(16:0/22:6(4Z,7Z,10Z,13Z,16Z,19Z)) | 1.64429695740997e-05 | 1.85296126499188e-05 | 1.82066138681808e-05 | 1.61972083420225e-05 | 5.45212184994042e-06 | 1.84277692530972e-05 | 1.94512799102411e-05 | 2.34547744232487e-05 | 2.73351384257198e-05 | 2.12539845509043e-05 | 2.67550951379954e-05 | 1.70754116550424e-05 | 1.55427159228766e-05 | 2.25542806838586e-05 | 1.45111580213996 | 0.624281365906121 | 0.0251985500463297 | 2.10874279394492 | up | -- |
| pos\_2280 | 3-(Acetyloxy)-2-hydroxypropyl octadecanoate | 8.62690083313899e-05 | 9.23350286377286e-05 | 9.23945627305991e-05 | 8.55313491336415e-05 | 8.34568719483747e-05 | 7.52077992612186e-05 | 8.81521630564025e-05 | 0.000101094981640989 | 9.92771469784058e-05 | 9.38895574334716e-05 | 0.000103255978579377 | 0.000125463319465811 | 8.58657700071587e-05 | 0.000101855524525743 | 1.18621802980688 | 0.24091902449878 | 0.0275947285837624 | 2.16678055500043 | up | -- |
| pos\_2327 | alpha-Micropteroxanthin B | 0.000364811882437993 | 0.000359595157956941 | 0.000351243318524951 | 0.000326871486159312 | 0.000368100283686745 | 0.000311723578216857 | 0.00045558082275077 | 0.000453446053916211 | 0.000444978046984619 | 0.000619918454619391 | 0.000438848304314384 | 0.000326987530007893 | 0.000347057617830466 | 0.000456626535432211 | 1.31570814750209 | 0.373720289430286 | 0.034402820533828 | 2.20934128660672 | up | -- |
| pos\_2339 | Catechin 3',5-diglucoside | 4.50392061465578e-06 | 1.1675506468171e-05 | 1.1101901745821e-05 | 8.87924902355009e-06 | 3.36663435082313e-06 | 1.02972308996771e-05 | 2.22236098715327e-05 | 1.75038428368262e-05 | 9.09706219838129e-06 | 2.19901734759324e-05 | 6.98394512962993e-06 | 1.85099037683498e-05 | 8.30407385044968e-06 | 1.60514228801087e-05 | 1.93295762648348 | 0.967802965876036 | 0.0346008349720682 | 2.19083099941769 | up | -- |
| pos\_2388 | Omega-Carboxy-trinor-leukotriene B4 | 7.58033909396618e-05 | 5.67611251367099e-05 | 6.68987874057057e-05 | 5.45547848160515e-05 | 5.63180481419547e-05 | 6.24494930929475e-05 | 8.83317074936292e-05 | 8.65664332553675e-05 | 9.54412918124529e-05 | 0.000147526780909741 | 0.000109232925816315 | 8.92508223399601e-05 | 6.21309382555052e-05 | 0.000102724993604578 | 1.65336298612029 | 0.708053406281612 | 0.00665983363938708 | 2.64392180216054 | up | -- |
| pos\_2448 | 2-Methyl-1-methylthio-2-butene | 2.31940270672947e-06 | 7.01353572411076e-06 | 2.81812203614476e-06 | 6.37512888430624e-06 | 2.10566181780601e-06 | 2.64541593276773e-06 | 6.13637922698417e-06 | 7.03885312964125e-06 | 9.81371104127437e-06 | 5.42534175502752e-06 | 6.05845625924512e-06 | 5.09400111598504e-06 | 3.87954451697749e-06 | 6.59445708802624e-06 | 1.69980188632141 | 0.907689694479036 | 0.0398742484879814 | 1.94751740851643 | up | -- |
| pos\_2482 | Thyrotropin releasing hormone | 4.48969499002613e-06 | 2.45501564951924e-06 | 5.04701545359071e-06 | 3.50657546396496e-06 | 3.53186567160188e-06 | 5.12188724210015e-06 | 4.55349934694708e-06 | 5.64773775873412e-06 | 5.94951286616722e-06 | 5.72494201785312e-06 | 5.48319817318222e-06 | 5.61722742569338e-06 | 4.02534241180051e-06 | 5.49601959809619e-06 | 1.3653545551763 | 0.489101389083814 | 0.0163812859712388 | 2.34930685907792 | up | -- |
| pos\_2554 | Melongoside O | 2.88703141514821e-06 | 1.33047655697517e-06 | 3.07997062670546e-06 | 1.80360182017325e-06 | 2.68822581672191e-06 | 1.44278422192959e-06 | 3.37934566747709e-06 | 3.26088871288975e-06 | 4.18133554812681e-06 | 6.57422552541767e-06 | 3.14348888086015e-06 | 2.48113420115545e-06 | 2.20534840960893e-06 | 3.83673642265449e-06 | 1.73974162356271 | 0.805887182143021 | 0.0421815433014541 | 2.10351835110341 | up | -- |
| pos\_2560 | DG(18:1(11Z)/22:5(7Z,10Z,13Z,16Z,19Z)/0:0) | 1.16357941017589e-06 | 1.94869876836947e-06 | 8.53022028763727e-06 | 4.29079676881982e-06 | 3.35700459685172e-06 | 3.18313477722567e-06 | 0 | 1.99472937528883e-06 | 1.59622090187457e-06 | 0 | 0 | 1.09662460070409e-06 | 3.74557243484664e-06 | 7.81262479644582e-07 | 0.208582931777308 | -12.4658673558716 | 0.0370390078295255 | 1.93521633561905 | down | -- |
| pos\_2565 | Phytosulfokine b | 1.31091222232293e-05 | 9.00809993142853e-06 | 1.28082555267511e-05 | 1.01549231861361e-05 | 1.21011420215864e-05 | 0 | 0 | 2.12273876301124e-06 | 8.84953248856816e-06 | 0 | 0 | 1.8192695533641e-07 | 9.53025714818857e-06 | 1.85903303448597e-06 | 0.195066408553238 | -10.044778936393 | 0.0126494874657795 | 2.28443689670523 | down | -- |
| pos\_2568 | Ramontoside | 0.000266394159727697 | 6.66342777296451e-05 | 0.000217729606293515 | 0.000129364256904878 | 0.000242644339159757 | 2.05332612217408e-07 | 2.85150101923268e-06 | 3.9964472938893e-06 | 8.26105194462058e-05 | 2.05633266207401e-06 | 2.92008604518797e-07 | 2.82703405727531e-06 | 0.000153828662071285 | 1.57723071805326e-05 | 0.102531654167438 | -3.98231590544878 | 0.0230374363220758 | 2.26820260796414 | down | -- |
| pos\_2587 | Lettucenin A | 0.00020274511850803 | 0.00021206175454169 | 0.000115657337407928 | 0.000150834257136382 | 8.99753318480094e-05 | 0.000194040976161546 | 0.000257801848493885 | 0.000223440614646888 | 0.000238965176817526 | 0.000200881166944013 | 0.000226628374829862 | 0.000203841299739229 | 0.000160885795933931 | 0.0002252597469119 | 1.40012202820195 | 0.547689388691955 | 0.0244277804076728 | 2.08472395774611 | up | -- |
| pos\_2622 | 4-(Hydroxymethyl)benzenediazonium(1+) | 0.000282023754933637 | 0.000171108038970938 | 0.000334610066031765 | 0.000232323787068686 | 0.000301122915944176 | 5.09358593701699e-05 | 0.000146576333712696 | 0.000110759982737507 | 0.000184742180379443 | 7.60022149223299e-05 | 7.21170875134866e-05 | 6.69713239542355e-05 | 0.000228687403719895 | 0.000109528187203283 | 0.478942807612776 | -0.951284004219696 | 0.0381703706273983 | 2.02199021404898 | down | -- |
| pos\_2660 | Isolinderanolide | 0.000708515080854387 | 0.000858095258630156 | 0.00110541648391961 | 0.000600578363215533 | 0.000967462485582657 | 0.00107242445931698 | 0.000788641314976542 | 0.000646529558374018 | 0.000629645824067759 | 0.000635516823561436 | 0.000659991114355796 | 0.000630607256323466 | 0.000885415355253221 | 0.000665155315276503 | 0.751235351103974 | -0.383678665672136 | 0.0434446997555266 | 2.08764812072352 | down | -- |
| pos\_268 | PYRITHYLDIONE | 6.90906509893135e-05 | 2.1986203658583e-05 | 6.39763751329079e-05 | 4.20297773678383e-05 | 0.00011982766901343 | 3.18871000396651e-06 | 4.55988558354288e-06 | 6.83679418527155e-06 | 1.82894832277181e-05 | 4.31025476324088e-06 | 4.99413679820928e-06 | 3.09099463268671e-06 | 5.33498976943399e-05 | 7.01359153177823e-06 | 0.131464010895795 | -2.55470049518722 | 0.0391246265238868 | 2.18306916459017 | down | -- |
| pos\_2712 | Capsianoside IV | 0.00239966783715559 | 0.0032660544839504 | 0.00267564377796986 | 0.00242457126650391 | 0.00215927681048513 | 0.00243532561413788 | 0.00177342837750843 | 0.00216475594622591 | 0.00189341800964783 | 0.00213195982153504 | 0.00222675373925636 | 0.00242878928105859 | 0.00256008996503379 | 0.00210318419587203 | 0.821527455908864 | -0.278927363589059 | 0.0364678994703437 | 1.97281067147547 | down | -- |
| pos\_2726 | Sorbic acid | 6.3111508135066e-06 | 2.64767465405844e-06 | 6.0007016439007e-06 | 4.25095002201264e-06 | 5.8069857317188e-06 | 3.38439851025723e-07 | 4.76183283719477e-07 | 1.00734504411042e-06 | 3.49093648047599e-06 | 5.03961231750085e-07 | 6.41862472184432e-07 | 4.98726197099223e-07 | 4.22598378603715e-06 | 1.10316911822327e-06 | 0.261044332888402 | -1.93308411834306 | 0.0214537807344293 | 2.19833290491741 | down | -- |
| pos\_279 | SWIETENINE | 0.00112740535689989 | 0.000925945331740467 | 0.000803101187826185 | 0.00112551830644579 | 0.00114900308621982 | 0.0008574299951002 | 0.000904091260710648 | 0.00141470900428277 | 0.00134770149088008 | 0.00180374941634016 | 0.00131907217601089 | 0.00140859114426971 | 0.000998067210705392 | 0.00136631908208238 | 1.36896500298484 | 0.43926667701605 | 0.02535261068733 | 2.21576943159448 | up | -- |
| pos\_2820 | 1,3-Dipropyl-8-cyclopentylxanthine [DPCPX] | 7.98718974633845e-05 | 5.97267321080274e-05 | 7.38761043451011e-05 | 6.38922409585642e-05 | 0.000111576349447508 | 1.82938802125939e-05 | 1.82915840133383e-05 | 2.35011687175363e-05 | 5.54491861195574e-05 | 1.86523252585342e-05 | 1.89038472740544e-05 | 2.31349307678286e-05 | 6.78728674225298e-05 | 2.63221736918082e-05 | 0.387815848827256 | -1.31415780195055 | 0.0188823126284416 | 2.30373823837444 | down | -- |
| pos\_2841 | Famciclovir | 4.20809602478822e-05 | 7.88257821141631e-05 | 7.79301196029486e-05 | 5.68241468993315e-05 | 6.25272210181293e-05 | 2.39634942807885e-05 | 1.32926293037135e-05 | 3.71409500485301e-05 | 4.41060027706796e-05 | 4.37400872640401e-05 | 2.67037312642742e-05 | 8.54379418460404e-06 | 5.70252873605405e-05 | 2.89211991393069e-05 | 0.507164461205668 | -1.11038076190996 | 0.0273605450200754 | 2.01390412040664 | down | -- |
| pos\_2850 | Sinapyl alcohol | 2.93402333035816e-06 | 4.24618997800066e-06 | 3.43483264302417e-06 | 3.22845288581625e-06 | 2.93895229296932e-06 | 1.65005767705157e-06 | 2.52576615315859e-06 | 1.88595495929847e-06 | 2.25276779062976e-06 | 2.07267745794393e-06 | 1.6094710083322e-06 | 1.99334726831587e-06 | 3.07208480120335e-06 | 2.0566641062798e-06 | 0.669468533379741 | -0.538493374088741 | 0.0313820371313533 | 2.09769678422171 | down | Metabolic pathways(ko01100) |
| pos\_286 | 4-(2,6,6-Trimethylcyclohexa-1,3-dienyl)but-3-en-2-one | 0.000226263214335516 | 0.000231908072866112 | 0.000260739605242808 | 0.000231656214243772 | 0.000223869503777332 | 0.000223623936807514 | 0.000252942355857194 | 0.000251825519588848 | 0.000282781261171758 | 0.000250450233844394 | 0.000249974214468431 | 0.0002388889438579 | 0.000233010091212176 | 0.000254477088131421 | 1.09212904388633 | 0.127280021466674 | 0.0275282678984926 | 2.11567655881831 | up | -- |
| pos\_311 | Cystophorene | 9.01350582426328e-06 | 4.4945035132473e-06 | 5.39339756696086e-06 | 4.84755146680013e-06 | 4.73260994421713e-06 | 4.57158799423378e-06 | 5.46666092858629e-06 | 6.76454078752787e-06 | 1.11178732637353e-05 | 1.52956772434734e-05 | 1.05482999746714e-05 | 8.3419296581653e-06 | 5.50885938495375e-06 | 9.58916364269326e-06 | 1.74068041542029 | 0.765633422686637 | 0.0374852821284731 | 2.11448808924858 | up | -- |
| pos\_321 | Pro Ile Leu Ile | 0.00556911675691724 | 0.00801961672800048 | 0.00633282176521621 | 0.00659965807494753 | 0.00699427692401941 | 0.00675468431022208 | 0.00624102113313941 | 0.00874545618890723 | 0.00730632563897749 | 0.00891936001060466 | 0.0100286944609159 | 0.0119955564809833 | 0.00671169575988716 | 0.00887273565225466 | 1.32198120559682 | 0.379767871015727 | 0.0478238297640925 | 2.00589197557294 | up | -- |
| pos\_340 | Norchelerythrine | 1.24616294460533e-05 | 1.78111673324469e-05 | 1.08011694625764e-05 | 1.46620134766884e-05 | 2.67274942059776e-06 | 2.69469706878023e-05 | 3.25493897258705e-05 | 8.16324112558009e-05 | 2.18809219421123e-05 | 2.60189936031034e-05 | 2.85088338395626e-05 | 3.74389114702975e-05 | 1.42259499710275e-05 | 3.80049103061245e-05 | 2.67152003089601 | 1.5528156039649 | 0.0456577947768966 | 1.97102475713456 | up | -- |
| pos\_367 | Thr Asn Thr Asn | 0.000260805404975882 | 0.00020961996475486 | 0.000255449165180804 | 0.000233774257675022 | 0.000216097617504226 | 0.000184437181435312 | 0.000265373558087726 | 0.000281453948663418 | 0.000229051504061019 | 0.000271818036148036 | 0.000260038800660341 | 0.000307218494405299 | 0.000226697265254351 | 0.000269159057004306 | 1.18730614902793 | 0.252252304919809 | 0.0234555543526371 | 2.21899028706231 | up | -- |
| pos\_375 | Isobutyrylmallotochromene | 8.6900281123637e-05 | 0.000136197273282615 | 0.000125720434885379 | 0.000136383312035498 | 0.000195677340198524 | 9.40998399727389e-05 | 4.88696830581599e-05 | 8.04176241587173e-05 | 8.59308028173874e-05 | 5.47712094406931e-05 | 8.30628216582262e-05 | 0.000128650303897588 | 0.000129163080249732 | 8.0283740838462e-05 | 0.621568800335486 | -0.706782648570274 | 0.0340893610254241 | 2.04760570926119 | down | -- |
| pos\_39 | Choline | 0.000138778474589391 | 5.32014466010217e-05 | 6.82317648693005e-05 | 5.45711169400529e-05 | 6.6613822984998e-05 | 4.68878999231789e-05 | 7.3165985784274e-05 | 0.000102767951404759 | 0.000219023871879198 | 0.000142133806240331 | 0.000228914691663387 | 0.000305516516127399 | 7.13807543179905e-05 | 0.000178587137183225 | 2.50189478788142 | 1.26246776053475 | 0.0291236696245985 | 2.13536737479058 | up | Choline metabolism in cancer(ko05231);;ABC transporters(ko02010);;Bile secretion(ko04976);;Metabolic pathways(ko01100);;Cholinergic synapse(ko04725);;Glycine, serine and threonine metabolism(ko00260);;Glycerophospholipid metabolism(ko00564) |
| pos\_488 | 3,4,5,6-Tetrahydrohippuric acid | 3.05201500534651e-05 | 4.49337430873169e-05 | 4.09865495504691e-05 | 2.64886035950766e-05 | 6.98920634425824e-05 | 1.49808863385266e-05 | 1.74165335741013e-05 | 1.85321853357624e-05 | 2.30889065246238e-05 | 1.62246274066655e-05 | 1.29083499541467e-05 | 1.54005902531664e-05 | 3.79669993445728e-05 | 1.72618655080777e-05 | 0.454654457978523 | -1.00319488197702 | 0.0434265240444731 | 2.17551177680436 | down | -- |
| pos\_496 | Rishitin | 5.26087361127946e-05 | 1.99393218821838e-05 | 3.63992231095985e-05 | 4.020931612617e-05 | 9.59271293881556e-05 | 2.8440212379531e-06 | 1.30798496820286e-06 | 6.20527752725508e-06 | 2.91206373639248e-05 | 1.37009820625277e-06 | 1.74123443259143e-06 | 2.80351544625248e-06 | 4.13212913094759e-05 | 7.09145799074657e-06 | 0.171617530963277 | -3.00257154815438 | 0.046139813644043 | 2.09180734798672 | down | -- |
| pos\_529 | 5-Aminopentanal | 1.28158077133298e-05 | 1.80480378755745e-05 | 1.51010737266287e-05 | 1.4635777320698e-05 | 1.14918674895642e-05 | 1.75181696797191e-05 | 1.56852530727645e-05 | 1.80747672969931e-05 | 1.58653021741188e-05 | 1.93788425786964e-05 | 1.94310263635561e-05 | 2.53674031350223e-05 | 1.49351223009191e-05 | 1.89670991035252e-05 | 1.26996610549068 | 0.34361650161323 | 0.0497012858425375 | 1.9716120599139 | up | Lysine degradation(ko00310);;Metabolic pathways(ko01100) |
| pos\_555 | Jasmolone | 1.98181849127965e-05 | 2.84540769012029e-05 | 2.10681297640056e-05 | 2.46588841458642e-05 | 2.11505586656537e-05 | 3.07522727359741e-05 | 2.75778735503238e-05 | 3.12814534739904e-05 | 2.77020699590005e-05 | 2.6480720513762e-05 | 3.01887878699911e-05 | 3.7727886926487e-05 | 2.43170178542495e-05 | 3.01597987155925e-05 | 1.24027538641306 | 0.319896711491484 | 0.0402625699736694 | 1.91579879195932 | up | -- |
| pos\_568 | 2-Butyl-3-phenyl-2-propen-1-al | 4.18706050138836e-06 | 6.14079959949648e-06 | 3.9998945085757e-06 | 4.3388882713076e-06 | 3.96649900950669e-06 | 6.32515563302387e-06 | 5.86413475857289e-06 | 7.19721488191482e-06 | 6.05132604814639e-06 | 5.56199549939458e-06 | 6.47614868526963e-06 | 8.87040069612297e-06 | 4.82638292054978e-06 | 6.67020342823688e-06 | 1.38202946969592 | 0.477621238180117 | 0.0205685651492742 | 2.07487395600203 | up | -- |
| pos\_578 | 2-Methyl-2-cyclopenten-1-one | 5.23913205674296e-05 | 2.40572886170949e-05 | 5.14476343591422e-05 | 3.63520918933274e-05 | 5.17078771364461e-05 | 2.64599744654588e-06 | 1.95384253080134e-06 | 8.2105151227664e-06 | 2.83574841912859e-05 | 2.55969856939223e-06 | 2.82106061566809e-06 | 3.7919636745499e-06 | 3.64337016699977e-05 | 7.94909411741064e-06 | 0.218179700471021 | -2.4433633187308 | 0.0160291346715477 | 2.27697152657639 | down | -- |
| pos\_58 | Gln Val Asn | 9.29616098739696e-05 | 7.45264970293775e-05 | 0.000109645752526162 | 7.6248103928916e-05 | 0.000149380036394272 | 1.24523210446998e-05 | 3.05503657513902e-05 | 3.07704137044168e-05 | 8.7587345736383e-05 | 1.70274486456102e-05 | 1.44738441518101e-05 | 1.14235651718216e-05 | 8.58690534662328e-05 | 3.19721638602386e-05 | 0.372336279132405 | -1.47673646276981 | 0.0374389261101259 | 2.05754693612778 | down | -- |
| pos\_605 | Trifolirhizin | 0.000115697510289205 | 5.96786090692995e-05 | 0.000150167139763978 | 9.55602434386027e-05 | 0.000187985353090952 | 2.77189881191008e-06 | 3.34088765328241e-06 | 1.78207061470256e-05 | 9.75866835780976e-05 | 2.27846143645199e-06 | 2.94989121588585e-06 | 1.36235545961718e-05 | 0.000101976792410658 | 2.29333641044859e-05 | 0.224888070730189 | -2.76106526460363 | 0.0336975622134517 | 2.0677747733844 | down | -- |
| pos\_644 | Isomasticadienonalic acid | 0.000261987855786794 | 0.000259169148353744 | 0.000311564398622452 | 0.000284025523660952 | 0.000344122137731848 | 0.000284996955134949 | 0.000350342831665443 | 0.000396844013026753 | 0.000420750311279888 | 0.000400894763734309 | 0.000388889466742849 | 0.000404107008488973 | 0.00029097766988179 | 0.000393638065823036 | 1.35281193908437 | 0.440802125210372 | 0.000130540489553532 | 2.91161572982081 | up | -- |
| pos\_659 | Stearidonic acid | 1.23713590292549e-05 | 1.55587587260224e-05 | 1.52586066896035e-05 | 1.32086357406202e-05 | 1.13773937606671e-05 | 1.40714402639578e-05 | 1.41676100222133e-05 | 1.63811009868414e-05 | 1.60212715065971e-05 | 1.55471697304735e-05 | 1.67875201867441e-05 | 1.87447260724773e-05 | 1.36410323683543e-05 | 1.62748997508911e-05 | 1.19308416778242 | 0.258394575970849 | 0.0160375336734266 | 2.26371534614015 | up | alpha-Linolenic acid metabolism(ko00592) |
| pos\_663 | Myristic acid | 1.29513039772099e-05 | 1.76437909442251e-05 | 1.32664465124293e-05 | 1.52016725454366e-05 | 1.4310497691325e-05 | 1.79509990813566e-05 | 1.77701415678675e-05 | 1.77189415746886e-05 | 1.59444122389141e-05 | 2.02035645639391e-05 | 1.96319659838073e-05 | 1.93326342918752e-05 | 1.52207851253304e-05 | 1.84336100368486e-05 | 1.21108141827529 | 0.283540548342996 | 0.0159403278094361 | 2.23456338400106 | up | Fatty acid biosynthesis(ko00061);;Metabolic pathways(ko01100) |
| pos\_692 | IV2Fuc-nLc4Cer | 9.95166805328634e-06 | 6.01434273972654e-06 | 9.11945774897101e-06 | 1.03479965469292e-05 | 8.81833752087605e-06 | 4.23148129084309e-06 | 9.46399646787797e-06 | 1.60331745476873e-05 | 1.25629335967814e-05 | 1.44900264312689e-05 | 1.89726938402228e-05 | 1.36372213968164e-05 | 8.08054731677204e-06 | 1.41933410467758e-05 | 1.75648263544179 | 0.847269240007787 | 0.0045085698957571 | 2.51995537710586 | up | -- |
| pos\_698 | 3-keto Fusidic acid | 0.000869909947417332 | 0.000781198008155487 | 0.000813961323438916 | 0.00089155780015881 | 0.000991050547480695 | 0.000769010672484456 | 0.000791905141920287 | 0.00132928474183864 | 0.00114388790406267 | 0.00158084470483485 | 0.00133638894257461 | 0.00136988322696543 | 0.000852781383189283 | 0.00125869911036608 | 1.47599271651396 | 0.535474550932628 | 0.0122596348600456 | 2.50104583067297 | up | -- |
| pos\_736 | 2-Methylpropyl formate | 9.55484734785898e-05 | 5.85902738638851e-05 | 0.000101917000749561 | 7.95304484676048e-05 | 9.33700879949189e-05 | 8.04430295528371e-06 | 6.51235243010823e-06 | 2.40885191279982e-05 | 7.40526928333921e-05 | 5.70906927610043e-06 | 8.16954530371788e-06 | 1.1595345672377e-05 | 7.28334312516406e-05 | 2.1687920773949e-05 | 0.297774255602718 | -2.0675623888655 | 0.018911361998892 | 2.16855198388131 | down | -- |
| pos\_742 | N-acetyl-5-methoxykynuramine | 1.6642613264265e-05 | 2.07909980478647e-05 | 1.8589651326806e-05 | 1.62005907049012e-05 | 1.62643856475737e-05 | 1.0782355748511e-05 | 1.11417806322752e-05 | 1.22014310825296e-05 | 1.4595352741914e-05 | 1.38443909185062e-05 | 1.26714265047982e-05 | 1.079001269186e-05 | 1.65450991233203e-05 | 1.25407324286472e-05 | 0.757972638010435 | -0.380443252864998 | 0.0316464364744542 | 2.0752440639174 | down | -- |
| pos\_760 | 6beta-hydroxybudesonide | 1.37919495749052e-05 | 1.58814542573125e-05 | 2.03926340514541e-05 | 1.43411047097191e-05 | 1.32690845071985e-05 | 1.08062389123216e-05 | 1.72148863322547e-05 | 2.12788302416365e-05 | 2.60256002751565e-05 | 2.85954473590035e-05 | 2.07789572534062e-05 | 1.6478917485083e-05 | 1.47470776688185e-05 | 2.17287731577567e-05 | 1.47342908511972 | 0.55753834983064 | 0.016362264265425 | 2.34390749377202 | up | -- |
| pos\_818 | Avocadene 1-acetate | 2.6293813054112e-05 | 1.83153903418727e-05 | 2.23190875477985e-05 | 2.19862742659369e-05 | 1.54324823277126e-05 | 1.78498786555006e-05 | 2.46724068803385e-05 | 2.49916228113165e-05 | 2.19012754888708e-05 | 2.51285978256046e-05 | 2.55559780826444e-05 | 2.77120968425034e-05 | 2.03661543654889e-05 | 2.49936629885464e-05 | 1.22721563138591 | 0.314053228331325 | 0.0338970377162651 | 2.18820627228169 | up | -- |
| pos\_83 | 8Z,11Z,14Z-heptadecatrienoic acid | 6.08211266368244e-05 | 9.97165066872565e-05 | 6.48127336591441e-05 | 8.84420071811391e-05 | 0.000109180432297386 | 0.000119142848218602 | 0.000119033449686916 | 0.000125166949184939 | 9.18186445404411e-05 | 0.000136803277041327 | 0.000133553408260513 | 0.000150647820531752 | 9.0352609113392e-05 | 0.000126170591540981 | 1.39642443952712 | 0.509611817793731 | 0.0182425931386949 | 2.14972884035012 | up | alpha-Linolenic acid metabolism(ko00592) |
| pos\_84 | GATIFLOXACIN | 6.38969723660283e-05 | 7.57378118982191e-05 | 0.000115119789983944 | 8.39768884581726e-05 | 0.000182017368520405 | 2.06267576056499e-05 | 2.73129974765128e-05 | 2.1694430647646e-05 | 6.54968220420819e-05 | 1.02688430477963e-05 | 7.16620742177369e-06 | 1.33932084155007e-05 | 9.02292648054032e-05 | 2.42220848418852e-05 | 0.268450428961433 | -2.02749077277121 | 0.0301165086143389 | 2.17990322594721 | down | -- |
| pos\_840 | DG(20:3(5Z,8Z,11Z)/22:6(4Z,7Z,10Z,13Z,16Z,19Z)/0:0) | 0.000646535397121394 | 0.000418345758336657 | 0.000521113807433651 | 0.000390644369507376 | 0.000371854884686684 | 0.000470635162790533 | 0.000459049920378506 | 0.000505268756219604 | 0.000963338498961197 | 0.000986370561627485 | 0.00116705103356387 | 0.00112196069131797 | 0.000469854896646049 | 0.000867173243678105 | 1.84561925366368 | 0.817398709311464 | 0.0237634903263255 | 2.27451548401422 | up | -- |
| pos\_849 | 24-Oxo-1alpha,25-dihydroxyvitamin D3 | 0.00563322002512106 | 0.00680019615160024 | 0.0059265042038135 | 0.00539566084616787 | 0.00564252252358959 | 0.00611577358430982 | 0.00683509475704201 | 0.00736855631143196 | 0.00737456378030664 | 0.00934771530679631 | 0.00766220717552571 | 0.00760723898602265 | 0.00591897955576701 | 0.00769922938618755 | 1.30076972113985 | 0.37654255117644 | 0.00229734766396928 | 2.70067882273218 | up | -- |
| pos\_866 | N-Oleoylethanolamine | 7.13415733671289e-05 | 9.55386692036802e-05 | 0.000130956325831409 | 6.74589033685474e-05 | 0.000102814196232674 | 0.000118175052445121 | 8.91995288084702e-05 | 6.31495034349558e-05 | 5.58449205575878e-05 | 6.22681335639282e-05 | 6.99830976775714e-05 | 5.9759937705342e-05 | 9.77141200747601e-05 | 6.67008536246426e-05 | 0.682612232230208 | -0.526459825447564 | 0.0288768140035416 | 2.12794833478974 | down | -- |
| pos\_963 | Permetin A | 1.08806344507762e-06 | 8.88687809860171e-07 | 1.76019251121238e-06 | 1.01635341164324e-06 | 3.34582456441859e-06 | 5.38043283148596e-07 | 2.76889810024298e-06 | 9.90608485694238e-06 | 2.27011202466147e-05 | 2.17342958792564e-05 | 1.14213360102459e-05 | 0 | 1.43952750422677e-06 | 1.14219558488837e-05 | 7.93451727413778 | -1.11163135882397 | 0.0478971389195755 | 2.08382489198841 | down | -- |
| pos\_996 | Capsianoside II | 2.65858342634618e-05 | 3.27219931280479e-05 | 2.98400084409965e-05 | 3.97255469237579e-05 | 2.51709961583501e-05 | 3.67868619204118e-05 | 3.58159957218388e-05 | 3.69918935214519e-05 | 4.20850143521857e-05 | 3.58800184221811e-05 | 4.66174089580093e-05 | 4.83479952436867e-05 | 3.18052068058377e-05 | 4.09563877032256e-05 | 1.28772587310164 | 0.373239835514628 | 0.0186528198974223 | 2.2213734806001 | up | -- |
